# The evaluation of locoregional tumoral involvement in the cooccurrence of hashimoto thyroiditis with papillary thyroid cancer: a case controlled study

**DOI:** 10.1186/s12902-023-01322-5

**Published:** 2023-03-24

**Authors:** Shirzad Nasiri, Seyed Mostafa Meshkati Yazd, Mahsa Gholami, Sepehr Shahriarirad, Sina Sharghi, Reza Shahriarirad

**Affiliations:** 1 Department of Surgery, Tehran University of Medical Sciences, Tehran, Iran; 2 Student research committee, School of Medicine, Shiraz University of Medical Sciences, Shiraz, Iran; 3 Department of Biostatistics and Data Science, School of Public Health, The University of Texas Health Science Center at Houston (UTHealth), Houston, TX 77030 USA; 4 Department of General Surgery, Alborz University of Medical Sciences, Alborz, Iran; 5 School of Medicine, Shiraz University of Medical Sciences, Shiraz, Iran; 6 Thoracic and Vascular Surgery Research Center, Shiraz University of Medical Science, Shiraz, Iran

**Keywords:** Papillary thyroid cancer, Hashimoto’s thyroiditis, cancer, Pathology, Surgery

## Abstract

**Background:**

Papillary thyroid carcinoma PTC is the most prevalent of all thyroid carcinomas. On the other hand, Hashimoto’s thyroiditis (HT), as part of the spectrum of autoimmune thyroid diseases, is a major cause of thyroid hypofunction worldwide. Several studies have aimed to indicate a possible correlation between PTC and HT over the years. This study aims to investigate the correlation between HT disease and PTC tumor invasion rate.

**Method:**

In the present cross-sectional study, PTC patients with HT were selected among patients referred to the surgical ward of Shariati hospital from 2016 to 2019 and compared in terms of tumor invasion and central LN dissection. Also, a similar group of PTC patients without HT undergoing total thyroidectomy was selected for comparison. The tumor invasion rate was assessed based on invasion indices obtained from postoperative permanent pathology specimens. These indices included tumor type and size, number of involved LNs, lymphovascular involvement, perineural involvement, thyroid capsule involvement, multifocal or unifocal tumor, extrathyroidal proliferation, marginal status, and necrosis. Data were obtained and compared in the two groups with SPSS version 22.0 software.

**Results:**

Based on the postoperative pathology reports, 50 (56.2%) PTC patients with Hashimoto thyroiditis were compared against 39 PTC patients without Hashimoto thyroiditis. No significant difference was found between the two groups regarding tumor invasion factors such as multifocality, lymphovascular invasion, marginal invasion, extrathyroidal invasion, capsular invasion, and necrosis.

**Conclusion:**

HT could not be mentioned as an aggravating factor of PTC invasion based on the invasion factors evaluated in pathology specimens.

## Introduction

Thyroid cancers are one of the most common types of endocrine malignancies. The most common type of malignancy in the thyroid gland is papillary thyroid cancer (PTC), with a higher prevalence among children and females [1–3]. PTC management includes a broad spectrum of options, from active surveillance to radical surgery and subsequent radioactive iodine (RAI) ablation. The prognosis of PTC with appropriate treatment is favorable, with a 10-year survival of more than 90%; however, recognizing the prognostic factors can play an essential role in reducing the dimensions of this cancer [4–6]. Several clinicopathologic characteristics have been recognized as being associated with an unfavorable prognosis, including older age, large primary tumor size, extrathyroidal extension (ETE), lymph node (LN), and distant metastasis [7–9].

Hashimoto’s thyroiditis (HT), also known as chronic lymphocytic or autoimmune thyroiditis, can be a leading cause of hypothyroidism [10–13]. Prevalence of HT is five to ten times more often in females than males, and its incidence increases with age, with a peak between 45 and 65 years [14]. It is significantly more common in patients with other autoimmune diseases [15]. Various factors, including genetic and non-genetic factors, play a major role in the development of this disease, but in many cases, no known risk factor is found in patients [12]. Also, produced anti-thyroid peroxidase (Anti-TPO) and thyroglobulin autoantibodies are responsible for the patients’ clinical symptoms [16].

A link between cancer and inflammation has been recognized for over a century. As early as 1863, Rudolf Virchow noted leukocytes in neoplastic tissue and suggested that this reflected the origin of cancer at sites of chronic inflammation [17]. Transforming normal cells to cancerous ones is a complex process that depends on various factors, including the interaction of environmental triggers, genetic transformations, and immune modulation. Some inflammatory diseases has been proven to be linked with cancer [18, 19]. According to some studies, one of the thyroid diseases that increases the risk of thyroid malignancy is HT, which increases the risk of thyroid cancer by up to 12 times, while in other studies, it was concluded that HT might be a protective factor for PTC because it is associated with less invasive disease at presentation and a lower recurrence rate [12, 20–22].

Overall, the association between tumor invasion in PTC and underlying HT is unclear. The present case-control study investigates this dilemma based on the postoperative pathology report and invasive indicants.

## Materials and methods

In the present study, PTC patients with HT were selected among patients referred to the surgical ward of Shariati hospital from 2016 to 2019 and compared in terms of tumor invasion and central LN involvement. Thyroglobulin (Tg) and anti-Tg levels were checked in all patients of our study. All patients also underwent Radioactive iodine (RAI) uptake scan and RAI therapy was administered based on ATA 2015 criteria [7, 23].

The typical histological features of Hashimoto’s thyroiditis include a lymphoplasmacytic infiltration, a germinal center formation, follicular destruction, a Hurthle cell change, and variable degrees of fibrosis [24, 25]. The HT diagnosis confirmed after surgery by permanent pathology, also pathology results were confirmed by two pathologists. Also, a similar group of PTC patients without HT undergoing total thyroidectomy was selected for comparison, which were recruited by random consecutive permanent pathology report and matched based on age and gender. The tumor invasion rate was assessed based on invasion indices obtained from postoperative permanent pathology specimens. These indices included tumor type and size, number of involved LNs, lymphovascular invasion (LVI), perineural involvement (PNI), thyroid capsule invasion (CI), multifocal or unifocal tumor, extrathyroidal extension (ETE), marginal status, and necrosis. According to AJCC Cancer Staging Manual, all PTC patients below 55 years of age with any T, any N and M0 are placed in stage 1. Based on this reference, the patients of this study are placed in stage 1 [26].

Metastasis or recurrence work up is done in follow-up after surgery with Tg measurement and RAI uptake scan [7, 23].

Data were obtained and compared in the two groups with SPSS version 22.0 software. The normality of data was evaluated with the Kolmogorov-Smirnov test. Data were reported as frequency and percentage (%), mean and standard deviation (SD), or median and quartiles. For data analysis, the Chi-square and Fisher’s exact test was used for categorical variables, while the independent sample t-test and Mann-Whitney U test for continues variables. A P-value of less than 0.05 was considered statistically significant.

## Results

Based on the postoperative pathology reports, 50 (56.2%) PTC patients with Hashimoto thyroiditis were compared with 39 PTC patients without Hashimoto thyroiditis. The features of the 89 patients in our study are demonstrated in Table 1. During the one-year follow-up of all patients, no cases of mortality and no cases of metastasis or recurrence based on RAI scan was reported.

**Table 1:** Features of papillary thyroid cancer patients based on Hashimoto thyroiditis

Variable		Total; N = 89	Hashimoto Thyroiditis		P-value*
			**Yes**; n = 50	**No**; n = 39	
**Gender**; n (%)	Male	13 (14.6)	5 (10.0)	8 (20.5)	0.228
	Female	76 (85.4)	45 (90.0)	31 (79.5)	
**Age (years)**; mean ± SD		38.08 ± 13.72	36.36 ± 11.57	40.28 ± 15.95	0.182
**Tumor type**; n (%)	Classic	59 (66.3)	35 (70.0)	24 (61.5)	0.402
	Other	30 (33.7)	15 (30.0)	15 (38.5)	
**Tumor size**; median [Q1 – Q3]		13 [8–24]	12.5 [8–20.25]	17 [5–30]	0.456
**Multifocality**	Multifocal	38 (42.7)	13 (33.3)	25 (50.0)	0.135
	Unifocal	51 (57.3)	25 (50.0)	26 (66.7)	
**Marginal invasion**	Yes	31 (34.8)	18 (36.0)	13 (33.3)	0.793
	No	58 (65.2)	32 (64.0)	26 (66.7)	
**Lymphovascular invasion**	Yes	11 (12.4)	6 (12.0)	5 (12.8)	1.000
	No	78 (87.6)	44 (88.0)	34 (87.2)	
**Perineural invasion**	Yes	4 (4.5)	2 (4.0)	2 (5.1)	1.000
	No	85 (95.5)	48 (96.0)	37 (94.9)	
**Extrathyroidal extension**	Yes	5 (5.6)	2 (4.0)	3 (7.7)	0.650
	No	84 (94.4)	48 (96.0)	36 (92.3)	
**Necrosis**	Yes	3 (3.4)	1 (2.0)	2 (5.1)	0.579
	No	86 (96.6)	49 (98.0)	37 (94.9)	
**Capsular invasion**	Yes	25 (28.1)	15 (30.0)	10 (25.6)	0.813
	No	64 (71.9)	35 (70.0)	29 (74.4)	
**Central LN involvement**; median [Q1 – Q3]		0 [0–1.5]	0 [0–1]	0 [0–2]	0.227

LN: Lymph node; SD: Standard deviation

*Chi-square/Fisher’s exact test or Mann-Whitney U/Independent sample t-test

**Fig. 1:**
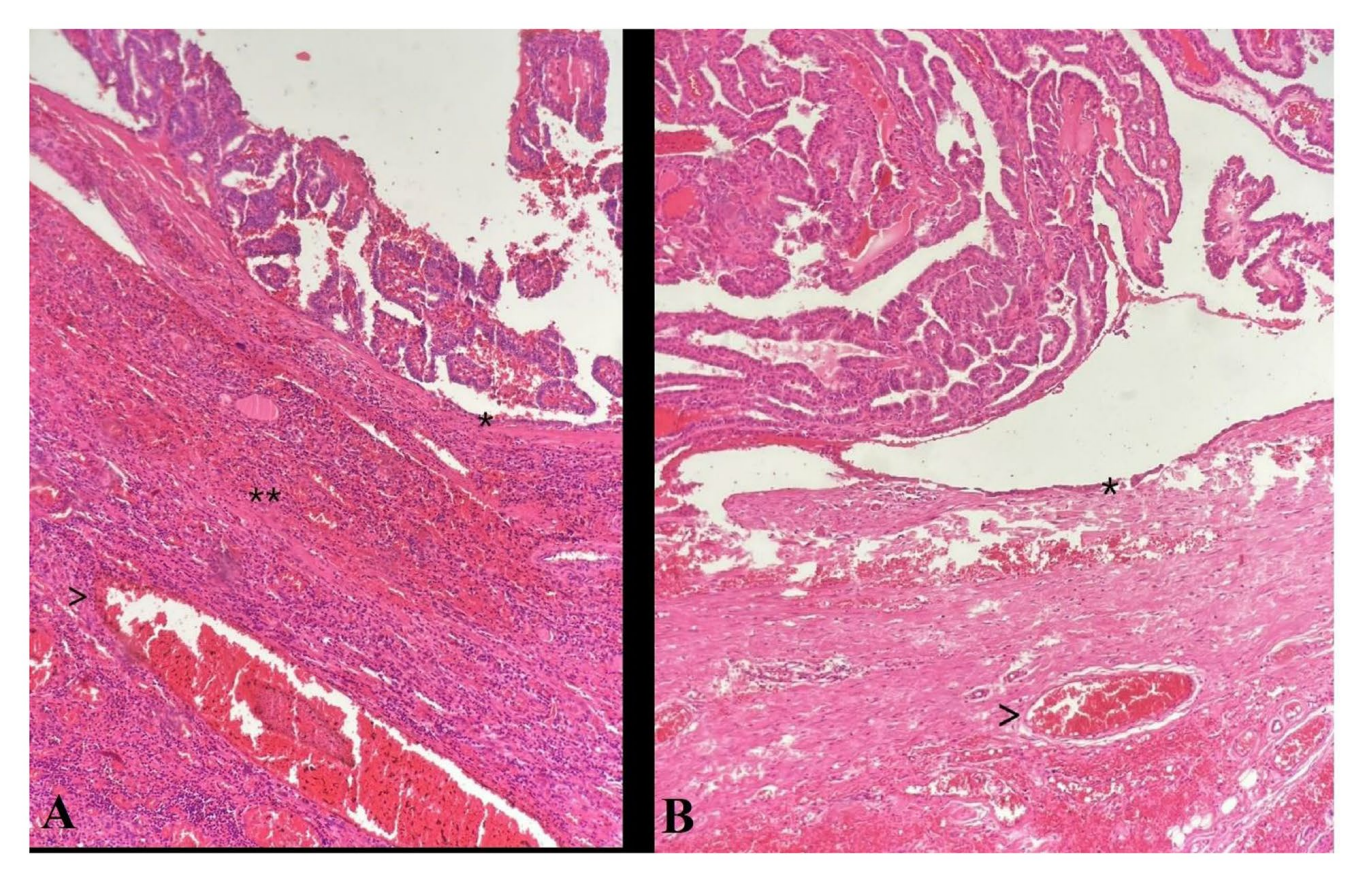
Photomicrograph of Papillary Thyroid Carcinoma with no capsular (*) nor vascular (arrow head) invasion, lymphovascular invasion was not identified; **A**: characterized with lymphocytic infiltration, indicating Hashimoto’s thyroiditis (**); **B**: Without thyroiditis background (hematoxylin and eosin x40)

As demonstrated in Table 1, there was no significant difference between the demographical and clinical variables of PTC patients with and without Hashimoto thyroiditis. The permanent pathology of the patients was also evaluated for the confirmation of diagnosis, in which Fig. 1 demonstrates a sample of the groups in our study with (Fig. 1A) and without (Fig. 1B) Hashimoto thyroiditis.

## Discussion

HT is the most common form of autoimmune thyroid disease, with an approximate annual incidence of 0.3 to 1.5 cases per 1000 persons [27, 28]. HT is widely considered to be associated with thyroid cancers and the development of thyroid nodules [29]. PTC is one of the most common endocrine malignancies, and the incidence of this disease has rapidly increased in recent years [30]. Evaluation of the relation of HT background and tumor invasion in patients with PTC has been a subject yielding mixed and contradicting results [12, 31, 32]. Although many inflammatory diseases have been proven to predispose to mutations and the progression of cells to cancer [33], this are still limited reports and an unclear conclusion about HT and PTC, which is the most common thyroid cancer. In this study, the invasion factors of PTC in HT patients and those without HT background were evaluated, and it was concluded that there is no significant difference in all tumor invasion indices investigated in our study between the two groups.

LN involvement was one of the invasion indicators in our study that was not significantly different between the two groups under study. In other words, HT cannot be considered an influential factor in PTC LN involvement. Studies by Feng Zhu et al. and Zhang et al. align with our findings, demonstrating no significant difference regarding LN involvement in patients with PTC and HT backgrounds compared with the group without HT background [34, 35].

Tumor multifocality can be presented in PTC patients, and the prevalence of multifocality ranges from 18–87% [35, 36]. In the present study, we found that the correlation between HT background and PTC multifocality is insignificant, demonstrating that HT background is not a risk factor for PTC tumor invasion. However, not all studies have reached the same result. For example, in contrast to our study, Shuai Donget al. concluded that the rate of multifocality and capsular invasion is higher in patients with an HT background [37].

Tumor size is another risk factor for PTC invasion, which was evaluated in our study between the case and control groups. According to our result, there is no significant difference in tumor size between the two groups. In a meta-analysis conducted by Ju-Han Lee et al. same result has been proved and concluded that there is no significant difference in tumor size between patients with or without HT background [32].

Despite the evaluations conducted in different studies, the results of similar studies are not entirely identical. Based on our results, it is not possible to count HT as an underlying disease that increases the rate of PTC invasion. Studies with larger sample sizes and future meta-analyses are likely to elucidate this association further. Also, studies based on genetic analysis in patients with PTC and HT will better illustrate this relationship.

We acknowledge that there are several limitations to our study. The present study is cross-sectional, while prospective multicentral studies with higher sample size and future follow-ups could provide more valuable evidence. Also, in the present study, genetic studies and studies on the genes of patients in the two groups were not performed. Therefore, studies based on genetic analysis in patients with PTC and HT are recommended because the genetic analysis will better determine this relationship.

## Conclusion

In conclusion of the present study, which was performed on 89 patients with PTC, HT could not be mentioned as an aggravating factor of PTC invasion because the invasion factors evaluated in pathology specimens such as multifocality, lymphovascular invasion, extrathyroidal invasion, perineural invasion, capsular invasion, and necrosis were not significantly different between case and control group. Overall, the association between HT and PTC remains unclear, and more prospective studies are needed to support or disprove this association; until then, HT patients do not need more clinical follow-ups rather than patients without HT.

## Data Availability

The datasets used and/or analyzed during the current study are available from the corresponding author on reasonable request and with permission of the Research Ethics Committee of the School of Medicine-Iran University of Medical Sciences.

## Abbreviations

PTC: Papillary thyroid cancer
HT: Hashimoto’s thyroiditis
RAI: Radioactive iodine
LN: Lymph node
ETE: Extrathyroidal extension
AITD: Autoimmune thyroid diseases
LVI: Lymphovascular involvement
PNI: Perineural involvement
CI: Capsule involvement

## References

[1] Brown RL, de Souza JA, Cohen EE (2011). Thyroid cancer: burden of illness and management of disease. J Cancer.

[2] Aschebrook-Kilfoy B, Schechter RB, Shih Y-CT, Kaplan EL, Chiu BC-H, Angelos P, Grogan RH (2013). The clinical and economic burden of a sustained increase in thyroid cancer incidence. Cancer Epidemiol Prev Biomarkers.

[3] Sosa JA, Udelsman R (2006). Papillary thyroid cancer. Surg Oncol Clin.

[4] Kloos RT (2005). Papillary thyroid cancer: medical management and follow-up. Curr Treat Options Oncol.

[5] Sampson E, Brierley JD, Le LW, Rotstein L, Tsang RW (2007). Clinical management and outcome of papillary and follicular (differentiated) thyroid cancer presenting with distant metastasis at diagnosis. Cancer: Interdisciplinary International Journal of the American Cancer Society.

[6] Zarebczan B, Chen H (2010). Multi-targeted approach in the treatment of thyroid cancer. Minerva chirurgica.

[7] Haugen BR, Alexander EK, Bible KC, Doherty GM, Mandel SJ, Nikiforov YE, Pacini F, Randolph GW, Sawka AM, Schlumberger M (2016). 2015 american thyroid Association management guidelines for adult patients with thyroid nodules and differentiated thyroid cancer: the american thyroid Association guidelines task force on thyroid nodules and differentiated thyroid cancer. Thyroid.

[8] Haddad RI, Nasr C, Bischoff L, Busaidy NL, Byrd D, Callender G, Dickson P, Duh Q-Y, Ehya H, Goldner W (2018). NCCN guidelines insights: thyroid carcinoma, version 2.2018. J Natl Compr Canc Netw.

[9] Craig SJ, Bysice AM, Nakoneshny SC, Pasieka JL, Chandarana SP (2020). The identification of intraoperative risk factors can reduce, but not exclude, the need for completion thyroidectomy in low-risk papillary thyroid cancer patients. Thyroid.

[10] Davies L, Welch HG (2006). Increasing incidence of thyroid cancer in the United States, 1973–2002. JAMA.

[11] Burgess JR, Tucker P (2006). Incidence trends for papillary thyroid carcinoma and their correlation with thyroid surgery and thyroid fine-needle aspirate cytology. Thyroid.

[12] DAILEY ME, LINDSAY S, SKAHEN R (1955). Relation of thyroid neoplasms to Hashimoto disease of the thyroid gland. AMA archives of surgery.

[13] Mazokopakis EE, Papadakis JA, Papadomanolaki MG, Batistakis AG, Giannakopoulos TG, Protopapadakis EE, Ganotakis ES (2007). Effects of 12 months treatment with L-selenomethionine on serum anti-TPO levels in patients with Hashimoto’s thyroiditis. Thyroid.

[14] Bossowski A, Otto-Buczkowska E. Schorzenia tarczycy o podłożu autoimmunologicznym.[W:] Pediatria–co nowego.Pod redakcją Ewy Otto-Buczkowskiej2007:108–120.

[15] Fallahi P, Ferrari SM, Ruffilli I, Elia G, Biricotti M, Vita R, Benvenga S, Antonelli A (2016). The association of other autoimmune diseases in patients with autoimmune thyroiditis: review of the literature and report of a large series of patients. Autoimmun rev.

[16] Fink H, Hintze G (2010). Autoimmune thyroiditis (Hashimoto’s thyroiditis): current diagnostics and therapy. Medizinische Klinik (Munich Germany: 1983).

[17] Wang Y, Wang W (2015). Increasing incidence of thyroid cancer in Shanghai, China, 1983–2007. Asia Pac J Public Health.

[18] Franks AL, Slansky JE (2012). Multiple associations between a broad spectrum of autoimmune diseases, chronic inflammatory diseases and cancer. Anticancer Res.

[19] Balkwill F, Mantovani A (2001). Inflammation and cancer: back to Virchow?. The lancet.

[20] Chen Y-K, Lin C, Cheng FT, Sung F, Kao C (2013). Cancer risk in patients with Hashimoto’s thyroiditis: a nationwide cohort study. Br J Cancer.

[21] Moon S, Chung HS, Yu JM, Yoo HJ, Park JH, Kim DS, Park YJ (2018). Associations between Hashimoto thyroiditis and clinical outcomes of papillary thyroid cancer: a meta-analysis of observational studies. Endocrinol metabolism.

[22] Dvorkin S, Robenshtok E, Hirsch D, Strenov Y, Shimon I, Benbassat CA (2013). Differentiated thyroid cancer is associated with less aggressive disease and better outcome in patients with coexisting Hashimotos thyroiditis. J Clin Endocrinol Metabolism.

[23] Randolph GW. Surgery of the thyroid and parathyroid glands e-book. Elsevier Health Sciences; 2020.

[24] Pearce EN, Farwell AP, Braverman LE (2003). Thyroiditis. N Engl J Med.

[25] Lloyd RV. Endocrine Pathology:: Differential diagnosis and molecular advances. Springer Science & Business Media; 2010.

[26] Edition S, Edge S, Byrd D. AJCC cancer staging manual. *AJCC cancer staging manual* 2017.

[27] Anil C, Goksel S, Gursoy A (2010). Hashimoto’s thyroiditis is not associated with increased risk of thyroid cancer in patients with thyroid nodules: a single-center prospective study. Thyroid.

[28] Randolph GW, Duh Q-Y, Heller KS, LiVolsi VA, Mandel SJ, Steward DL, Tufano RP, Tuttle ftATASACsToTCNS R (2012). The prognostic significance of nodal metastases from papillary thyroid carcinoma can be stratified based on the size and number of metastatic lymph nodes, as well as the presence of extranodal extension. Thyroid.

[29] Cipolla C, Sandonato L, Graceffa G, Fricano S, Torcivia A, Vieni S, Latteri S, Latteri MA (2005). Hashimoto thyroiditis coexistent with papillary thyroid carcinoma. Am Surg.

[30] Jung K-W, Won Y-J, Kong H-J, Oh C-M, Cho H, Lee DH, Lee KH (2015). Cancer statistics in Korea: incidence, mortality, survival, and prevalence in 2012. Cancer Res treatment: official J Korean Cancer Association.

[31] Liang J, Zeng W, Fang F, Yu T, Zhao Y, Fan X, Guo N, Gao X (2017). Clinical analysis of Hashimoto thyroiditis coexistent with papillary thyroid cancer in 1392 patients. Acta Otorhinolaryngol Ital.

[32] Lee J-H, Kim Y, Choi J-W, Kim Y-S (2013). The association between papillary thyroid carcinoma and histologically proven Hashimoto’s thyroiditis: a meta-analysis. Eur J Endocrinol.

[33] Shacter E, Weitzman SA (2002). Chronic inflammation and cancer. Oncol (Williston Park NY).

[34] Zhu F, Shen YB, Li FQ, Fang Y, Hu L, Wu YJ. The effects of Hashimoto thyroiditis on lymph node metastases in unifocal and multifocal papillary thyroid carcinoma: a retrospective Chinese cohort study.Medicine2016, 95(6). 10.1097/MD.0000000000002674 PMC4753890 26871795

[35] Zhang L, Li H, Ji Q-h, Zhu Y-x, Wang Z-y, Wang Y, Huang C-p, Shen Q, Li D-s, Wu Y (2012). The clinical features of papillary thyroid cancer in Hashimoto’s thyroiditis patients from an area with a high prevalence of Hashimoto’s disease. BMC Cancer.

[36] Zhang Y, Dai J, Wu T, Yang N, Yin Z (2014). The study of the coexistence of Hashimoto’s thyroiditis with papillary thyroid carcinoma. J Cancer Res Clin Oncol.

[37] Dong S, Xie X-J, Xia Q, Wu Y-J (2019). Indicators of multifocality in papillary thyroid carcinoma concurrent with Hashimoto’s thyroiditis. Am J cancer Res.

